# Neopterin and Beta-2 Microglobulin Relations to Immunity and Inflammatory Status in Nonischemic Dilated Cardiomyopathy Patients

**DOI:** 10.1155/2014/585067

**Published:** 2014-08-18

**Authors:** Celina Wojciechowska, Jan Wodniecki, Romuald Wojnicz, Ewa Romuk, Wojciech Jacheć, Andrzej Tomasik, Bronisława Skrzep-Poloczek, Beata Spinczyk, Ewa Nowalany-Kozielska

**Affiliations:** ^1^Second Department of Cardiology, School of Medicine with the Division of Dentistry, Medical University of Silesia, Ulica M.C. Skłodowskiej 10, 41-800 Zabrze, Poland; ^2^Department of Histology and Embryology, School of Medicine with the Division of Dentistry, Medical University of Silesia, 41-800 Zabrze, Poland; ^3^Department of Biochemistry, School of Medicine with the Division of Dentistry, Medical University of Silesia, 41-800 Zabrze, Poland

## Abstract

*Background*. The aim of the study was to assess the relationships among serum neopterin (NPT), *β*2-microglobulin (*β*2-M) levels, clinical status, and endomyocardial biopsy results of dilated cardiomyopathy patients (DCM).* Methods*. Serum NPT and *β*-2 M were determined in 172 nonischaemic DCM patients who underwent right ventricular endomyocardial biopsy and 30 healthy subjects (ELISA test). The cryostat biopsy specimens were assessed using histology, immunohistology, and immunochemistry methods (HLA ABC, HLA DR expression, CD3 + lymphocytes, and macrophages counts).* Results*. The strong increase of HLA ABC or HLA DR expression was detected in 27.2% patients—group A—being low in 72.8% patients—group B. Neopterin level was increased in patients in group A compared to healthy controls 8.11 (4.50–12.57) versus 4.99 (2.66–8.28) nmol/L (*P* < 0.05). *β*-2 microglobulin level was higher in DCM groups A (2.60 (1.71–3.58)) and B (2.52 (1.51–3.72)) than in the control group 1.75 (1.28–1.96) mg/L, *P* < 0.001. Neopterin correlated positively with the number of macrophages in biopsy specimens (*P* < 0.05) acute phase proteins: C-reactive proteins (*P* < 0.05); fibrinogen (*P* < 0.01); and NYHA functional class (*P* < 0.05) and negatively with left ventricular ejection fraction (*P* < 0.05).* Conclusions*. Neopterin but not *β*-2 microglobulin concentration reflected immune response in biopsy specimens. Neopterin correlated with acute phase proteins and stage of heart failure and may indicate a general immune and inflammatory activation in heart failure.

## 1. Introduction

Inflammatory dilated cardiomyopathy results from myocarditis induced by infectious and noninfectious triggers. Most of the evidence which suggests that myocarditis and inflammatory cardiomyopathy are autoimmune diseases come from animal models [1, 2]. The macrophages and T lymphocytes were found to be directly engaged in cell-mediated immune mechanism in experimental autoimmune myocarditis (EAM) [3–5]. Endomyocardial biopsy (EMB) is considered the diagnostic “gold standard” for myocarditis and both cells mentioned above might be assessed in it [6, 7]. To increase the diagnostic sensitivity EBM immunohistochemistry may be used for the identification of the inflammatory infiltration and for the detection of HLA-DR upregulation [8]. Progression from myocarditis to dilated cardiomyopathy (DCM) seems to occur predominantly in patients with histologically confirmed persistent (chronic) inflammation [9]. Besides cell-mediated myocytes damage, various cytokines including interleukin-1*β* (IL-1*β*), interleukin IL-2 (IL-2), interleukin IL-6 (IL-6), interferon-*γ* (IFN-*γ*), and tumor necrosis factor-*α* (TNF-*α*) have multiple biological activities and modulate immune response [10, 11].

Neopterin (NPT) arising from guanosine triphosphate (GTP) is mainly secreted by activated macrophages. IFN-*γ* and TNF-*α* were found to be the most frequent inducers of its synthesis [12]. Elevated NTP level was observed in infectious diseases, allograft rejection, disorders with potential autoimmune pathogenesis, and atherosclerosis [13–15]. Despite NPT being produced locally, serum level of NPT can reflect the extent and activity of disease. *β*-2 microglobulin (*β*-2M) is a light chain of HLA class I and its elevated serum concentration indicates cell surface expression of HLA. Increased level of *β*-2M was reported in viral infections and in chronic inflammatory and lymphoproliferative disorders [16, 17].

Accordingly, the aim of this study was to assess the usefulness of NPT and *β*-2M serum concentration as the potential biomarkers that may reflect upregulation of immune system in DCM patients.

## 2. Study Group and Methods

### 2.1. Patients

We recruited 172 patients hospitalized due to dilated cardiomyopathy and diagnosed according to the WHO criteria [18] who underwent endomyocardial biopsy. Chronic heart failure was recognized if dyspnea or fatigue at rest or on exertion in association with an ejection fraction (EF) ≤45% was present. All patients were clinically stable and received optimal conventional heart failure therapy including ACE inhibitors, *β*-blockers, digitalis, and diuretics for at least 6 months. Patients were excluded from the study if they had at least one the following: any changes in coronary vessels as assessed by coronary angiography, valvular disease except relative mitral regurgitation, connective tissue disease, endocrine disorders, renal insufficiency (estimated glomerular filtration rate using Modification of Diet in Renal Disease-MDRD formula <60 mL/min/1.73 m^2^), infectious disease, malignancy, moderate or severe hypertension, and alcohol abuse.

### 2.2. Clinical Assessements 

Noninvasive clinical assessment included the following: physical examination, ECG, and echocardiography. Echocardiographic images were acquired in standard views as recommended by the American Society of Echocardiography Committee. Left ventricular end-diastolic volume (EDV) and end-systolic volume (ESV) were obtained from the apical 4- and 2-chamber views by modified Simpson's method. Left ventricular ejection fraction (EF) was calculated in a standard manner as follows: EDV − ESV × 100/EDV to assess ventricular systolic function. The New York Heart Association (NYHA) classification was used to assess functional capacity. To rule out coronary artery disease all patients underwent coronary angiography.

### 2.3. Biochemical Methods

Blood samples for laboratory assessments were obtained from the patients at time of the biopsy. Serum was separated by centrifugation at 1500 g for 10 minutes and was frozen at −70°C and protected from light. Neopterin and *β*-2 microglobulin concentrations were determined in serum by ELISA method using commercially available kit manufactured by IBL, Hamburg (Germany). The limit of detection for neopterin was 0.7 nmol/L. The intra- and interassay coefficient of variation were <5.5% and <10.3%. Serum for *β*-2 microglobulin assay was diluted in a ratio of 1 : 50. The minimum detectable concentration of *β*-2 microglobulin was 0.1 mg/L. The intra- and interassay coefficient of variation were <11.6% and <15.5%. Additionally, we also determined serum creatinine, fibrinogen, and hs-CRP concentrations using routine techniques.

### 2.4. Endomyocardial Biopsy

At endomyocardial biopsy procedure a minimum of four specimens was obtained from the right side of the ventricular septum. Specimens were routinely distributed for histological, immunohistological, and immunohistochemical studies. Histopathological examination was performed by light microscopy according to Dallas classification [6] and immunohistologically as described previously [19]. For immunohistochemistry, frozen sections were incubated with murine monoclonal antihuman antibodies: anti-HLA class II (DR antigens), Alpha chain (clone TAL.1B5), anti-HLA class I (ABC antigens, clone W6/32), anti-CD3 for T lymphocytes (Clone T3-4B5), and antimacrophages (clone EBM11). The bound primary antibody was detected using the EnVision method (DAKO EnVision Kit/DAKO A/S). Each specimen was evaluated qualitatively and quantitatively for CD 3 lymphocytes and macrophages count and semiquantitatively for HLA expression on the previously defined and presented below immunoreactivity scoring system IR:

 0 lack of focal staining on the endothelial and interstitial cells,
 1+ focal staining on the endothelial and other interstitial cells,
 2+ multifocal staining restricted to interstitial cells,
 3+ diffuse endothelial and concomitant focal cardiac myocyte staining,
 4+ diffuse endothelial and myocyte staining [8].

IR ≥ 3+ was assessed as positive for major histocompatibility complex. For the purpose of this study patients were divided into two groups. Group A is patients with positive IR ≥ 3+ for HLA ABC or HLA DR, considered as having strong immune mediated inflammation. Group B is patients with weak immunoreactivity, staining of HLA complex IR of 0–2+. Group C is control group for neopterin and *β*-2 microglobulin assay comprised of healthy volunteers from hospital staff (8 male and 22 female, aged 39.03 ± 9.86 years). Approval of Local Ethic Committee was obtained.

### 2.5. Statistical Analysis

Normally distributed data are described as mean and SD, but nonnormally distributed data are presented as median and interquartile range. Frequencies of categorical variables were compared using Chi-square test with Yates correction (*χ* ^2^). Continuous variables between groups were compared using Student's* t*-test but nonnormally distributed data were compared using Mann-Whitney* U* test. A nonparametric Kruskal-Wallis* H* test was used to analyze differences of neopterin and *β*-2 microglobulin concentrations among examined groups of patients and post hoc Mann-Whitney* U* test was applied. Spearman's rank correlation coefficients (*r*) were computed for assessing mutual association among biochemical, biopsy and clinical results.

## 3. Results

The strong expression of HLA ABC or HLA DR (IR ≥ 3+) was detected in 46 patients—group A, low expression (IR 1 or 2+ for at least one HLA class) was indicated, respectively, in 123 patients—group B, and lack of expression (HLA ABC and HLA DR IR = 0) was detected in 3 patients who were excluded from analysis. Clinical characteristic of all DCM patients and groups A and B is presented in Table 1.

In patients with stronger HLA expression, lymphocytes CD3 and macrophages infiltrations were significantly more often presented in biopsy specimens. In group A 26.1% (12/46) patients were diagnosed as active myocarditis according to Dallas criteria in comparison to 6.5% (8/123) patients from group B (*P* < 0.001). As indicated, patients with stronger HLA expression presented worse functional capacity assessed by NYHA class (*P* < 0.01), but other clinical and echocardiographic parameters did not differ.

The concentrations of serum cell immune activation and inflammation markers are shown in Tables 2 and 3.

Neopterin concentration was increased in patients with strong activation of HLA molecule compared to the control group, *P* < 0.05. The difference in neopterin concentrations between DCM groups A and B was not significant (Table 2). On the other hand, among groups according to the Dallas criteria, neopterin level was higher in the group of patients with MCI (combined borderline and active) than in patients without MCI and control where the difference was statistically significant (Table 3).

*β*-2 microglobulin concentration was significantly higher in both DCM groups than in control, respectively: group A *P* < 0.001 and group B *P* < 0.00. Additionally *β*-2 microglobulin level was nonsignificantly elevated in patients with active myocarditis according to the Dallas criteria than in DCM patients without myocarditis.

**Figure 1:**
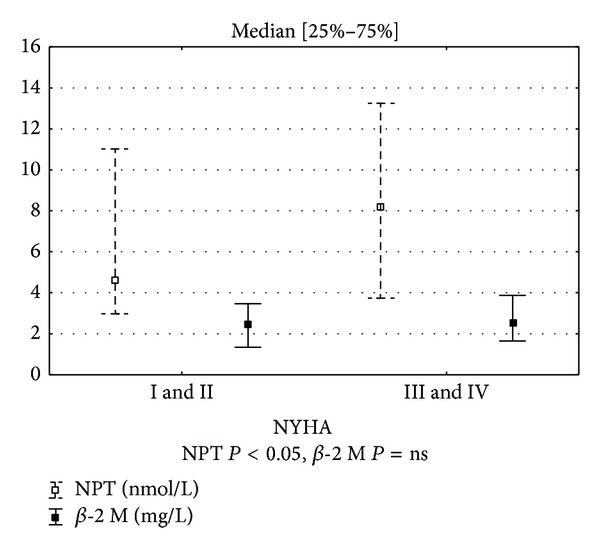
Neopterin and *β*-2 microglobulin concentrations in patients with NYHA classes I, II, III, and IV, median [25%–75%].

Higher serum neopterin levels were observed in patients with functional classes NYHA III and IV than in combined I and II NYHA functional classes (Figure 1).

Spearman's rank correlations among NPT and *β*-2 M levels and acute phase proteins and echocardiographic parameters in all DCM subjects are presented in Table 4.

**Figure 2:**
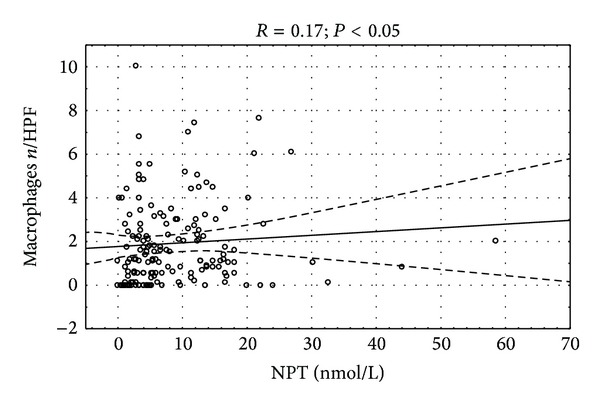
Correlation between serum neopterin concentration and macrophages in biopsy *R* = 0.17; *P* < 0.05.

**Figure 3:**
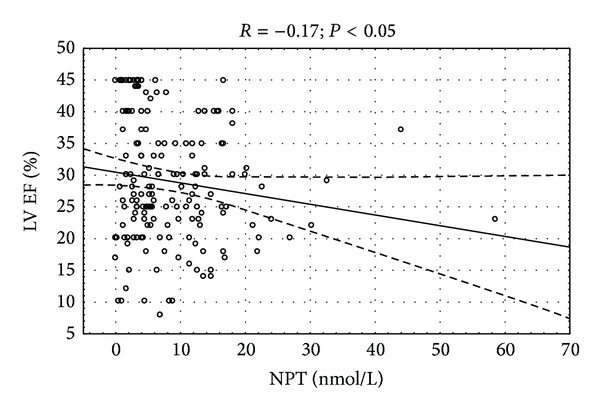
Correlation between serum neopterin and left ventricular ejection fraction *R* = (−0.17); *P* < 0.05.

**Figure 4:**
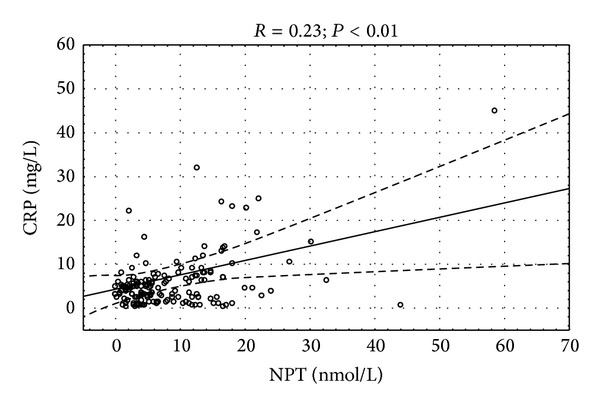
Correlation between serum neopterin and C-reactive protein concentration *R* = 0.23; *P* < 0.01.

**Figure 5:**
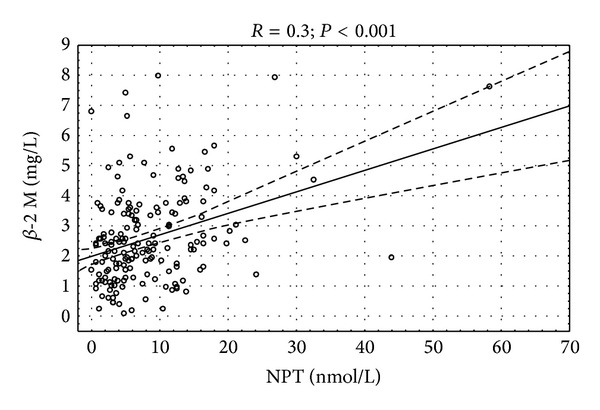
Correlation between serum neopterin and *β*-2 microglobulin concentration *R* = 0.3; *P* < 0.001.

Only neopterin concentration had significant correlation with count of macrophages in endomyocardial biopsy specimens (Figure 2). Significant correlation of left ventricular ejection fraction and NYHA class with neopterin (*P* < 0.05) and nonsignificant trend towards significance of *β*-2 microglobulin with ejection fraction (*P* = 0.09) were observed. There was positive correlation between serum concentration of cellular immune markers and acute phase proteins (Figures 3, 4, and 5).

## 4. Discussion 

Patients with heart failure due to DCM, especially if the conventional therapy is ineffective for them, need further diagnostic procedure including endomyocardial biopsy. Immune-mediated myocarditis may be identified in about one-third of DCM patients particularly in young adults and in cases of immune activation without presence of viruses immunosuppression therapy may be used [20]. Up to now no available serum markers have been acknowledged as helpful in diagnostic decision making and choice of the best treatment option [21]. We decided to assess whether serum NPT and *β*-2 M may be useful for diagnostic procedure in DCM patients because those parameters are valuable tools for detection, staging, and monitoring of other diseases [13, 22, 23]. Moreover, NPT and *β*-2 M are stable biomarkers of cellular immune activation, easy to measure, and not expensive. Some recent analyses have shown that serum levels of NPT are elevated also in patients with coronary artery disease and carotid and peripheral artery disease. The concentration of this molecule might predict adverse cardiovascular events in patients with coronary and carotid arteries disease. In addition, NPT level is related to the development of heart failure [24, 25]. The local and systemic inflammations play an important role in the development of atherosclerosis and are responsible for the instability in coronary artery disease. Therefore we intended to investigate the group of nonischemic cardiomyopathy to exclude influence of atherosclerosis on inflammatory markers. It should be emphasized that the biopsy procedure was performed at patients with chronic and clinically stable heart failure treated optimally for at least 6 months without complete recovery. It is relatively late according to current clinical practice guidelines [26] and partly explains the low percentage of active myocarditis. The increased concentrations of NPT are found in dilated cardiomyopathy group with strong immune activation—HLA expression in endomyocardium in comparison to controls, but the difference with the group of weak immune activation was not significant. Elevated NPT concentrations in patients with heart failure due to idiopathic or inflammatory DCM were described previously, especially in patients with active myocarditis assessed according to the Dallas criteria [27–29]. In our patients active myocarditis was diagnosed only in twenty patients and NPT concentration was increased in MCI group including active and borderline compared to controls. Caforio et al. did not find any differences either in mean value or in frequency of abnormal results of NPT among idiopathic cardiomyopathy, ischemic cardiomyopathy patients, and controls, but patients with myocarditis according to the Dallas criteria were excluded from the study [30].

Data concerning *β*-2 M concentrations in dilated cardiomyopathy are different. In keeping with our results Klappacher et al. found elevated *β*-2 M plasma level correlating with parameters of stimulated renin-angiotensin system and with T cellular hyperresponsiveness in DCM patients [31]. Caforio et al. observed the higher mean level and the proportion of abnormal results of *β*-2 M in ischaemic but not in idiopathic cardiomyopathy [30].

In our study we failed to find associations between serum NPT or *β*-2 M levels and HLA expression or lymphocytes CD3 count in endomyocardial specimens; only correlation of NPT with macrophages infiltrations was found. Similarly, correlations between serum NPT or *β*-2 M levels and HLA class I and II and ICAM-1 expression in patients with idiopathic non- inflammatory cardiomyopathy was not found[30]. Lack of correlations between concentration of *β*-2 M, light invariant chain of the class major histocompatibility molecule, and HLA I expression in immunohistological staining endomyocardium may be surprising. Some data obtained so far indicate that there is no simple correlation among HLA I complex on the surface of cells, its soluble form, and its part *β*-2 microglobulin in serum [32, 33]. Most of HLA class I molecules on the surface of cells are connected with presented antigens during interaction with T lymphocytes. Some molecules are not connected and therefore they may get into the blood stream. Serum NPT and *β*-2 M concentrations depend on their production in states of lymphocytes T stimulation and renal elimination. Previous studies have shown that serum level of NPT in group with creatinine clearance >60 mL/min was comparable to healthy control and significantly higher in group with creatinine clearance 10–60 mL/min [34]. *β*-2 M is an indicator of GFR, but some nonrenal factors were found independent of kidney function predictors of its concentration in healthy elderly (e.g., CRP level and white blood cells count) [35]. In patients with heart failure glomerular filtration may be decreased, so we excluded patients with eGFR <60 mL/min/1.73 m^2^.

We observed that increased NPT concentrations were associated with higher NYHA class. Similar observations of elevated NPT and/or *β*-2 M concentrations in dilated cardiomyopathy were noticed earlier [29–31]. In our DCM group like in previous data higher serum NPT and *β*-2 M concentrations correlated with lower left ventricular ejection fraction [30, 31]. The participation of inflammation and immune reactions in pathogenesis of heart failure was confirmed in clinical and experimental investigations. The concentrations of TNF-*α* and INF-*γ* were reported to be raised in patients with heart failure [36, 37]. Both are known stimulators of NPT synthesis, HLA expression, and turnover with *β*-2 M secretion into blood [38, 39]. The correlations between neopterin concentration and TNF-*α* or INF-*γ* in heart failure were found [40]. The mechanisms of immune activation and the place of cytokines, NPT, and *β*-2 M generation are not precisely established. Increased concentrations of cytokines may be associated with the immune reactions in cardiac tissue during viral infection and inflammation stage or the excessive load of the heart [41].

Beside the heart, their source may be other organs for example, intestines. Disturbances resulting from circulatory insufficiency such as hypoxia or congestion may cause inflammation by free radicals reactions and bacterial penetration through intestinal wall, therefore in some reports congestive heart failure is considered a state of chronic low-grade inflammation. The positive correlation between neopterin and acute phase proteins concentration indicated in our study might confirm these data. On the other hand, the experimental data indicated that NPT might be a pathogenic factor in the development of cardiac dysfunction in chronic disease states with high neopterin levels secondary to activation of the immune system [42].

The upregulation of HLA on cardiac tissue and their damage is a criterion which makes it possible to recognize active chronic myocarditis. Despite various data, concerning the effectiveness of immunosuppressive treatment, we can offer this therapy to patients with chronic myocarditis with increased HLA expression as an efficient method to improve the systolic function of left ventricle [19]. It is worth noting that an active inflammation and strong immune response were revealed in some cases of relatively long-lasting dilated cardiomyopathy.

Perhaps biopsy should be considered in dilated cardiomyopathy patients with elevated immune and inflammatory markers especially neopterin concentration.

## 5. Conclusion

Increased serum concentrations of neopterin reflect strong inflammatory-immunological response in endomyocardium of patients with dilated cardiomyopathy. *β*-2 microglobulin concentration is elevated in all patients with DCM independently on immune activation in myocardium.

Correlations between above markers of cellular immune activation with acute phase proteins level and left ventricle ejection fraction suggest the participation of low grade inflammatory state in pathophysiology of heart failure potentially related to its severity.

## Figures and Tables

**Table 1:** Clinical data of all dilated cardiomyopathy patients (DCM) and comparison between groups A and B.

	DCM *n* = 169	Group A *n* = 46	Group B *n* = 123	*P* A versus B
Sex M/F	133/36	40/6	93/30	ns
Age, y $\bar{X}$ (±SD)	43.2 (±10.4)	44.01 (±9.9)	42.9 (±10.5)	ns
Hypertension *n* (%)	49 (28.99)	13 (28.26)	36 (29.26)	ns
Diabetes mellitus *n* (%)	10 (5.91)	3 (6.52)	7 (5.69)	ns
Prior infection *n* (%)	33 (19.52)	10 (21.73)	24 (18.69)	ns
NYHA functional class I/II/III/IV *n* (%)	13/90/58/8 (8/53/34/5)	2/16/26/2 (4/35/57/4)	11/74/32/6 (9/60/26/5)	<0.05
ECG *n* (%)				
Normal	16 (9.4)	5 (10.8)	11 (8.9)	ns
LBBB	32 (18.9)	8 (17.3)	24 (19.51)	ns
RBBB	7 (4.14)	2 (3.34)	5 (4.06)	ns
Atrial fibrillation	37 (21.89)	11 (23.91)	26 (21.13)	ns
LV-overload	38 (22.48)	11 (23.91)	27 (21.95)	ns
UKG $\bar{X}$ ± SD				
EF (%)	29.67 ± 11.37	28.67 ± 11.27	30.04 ± 11.41	ns
LV EDV (mL)	189.81 ± 79.07	196.30 ± 76.77	187.39 ± 80.4	ns
LVESV (mL)	139.53 ± 68.72	144.43 ± 63.17	137.71 ± 70.8	ns
Left atrium (cm^2^)	21.96 ± 5.45	22.69 ± 5.30	21.69 ± 5.51	ns
Right ventricle (cm)	3.32 ± 0.59	3.39 ± 0.6	3.30 ± 0.59	ns
Biopsy				
CD3 (*n*/HPF)	1.04 ± 0.75	1.99 ± 0.78	0.68 ± 0.70	<0.01
Mac (*n*/HPF)	2.25 ± 2.01	3.31 ± 2.99	1.86 ± 1.65	<0.01
Dallas active *n* (%)	20 (11.8%)	12 (26.1%)	8 (6.5%)	<0.01
Dallas borderline	70 (41.4%)	22 (47.8%)	48 (39.0%)	ns
Dallas no MCI	79 (46.8%)	12 (26.1%)	67 (54.5%)	<0.01

**Table 2:** Serum concentrations of immune activation and inflammatory markers in all DCM patients, group A, group B, and control, median (25th–75th percentiles).

	All DCM *n* = 169	Group A *n* = 46	Group B *n* = 123	*P* A versus B	Group C *n* = 30	*P* A versus C∗ B versus C∗∗
NPT nmol/L	5.78 (3.27–12.48)	8.11 (4.50–12.57)	5.36 (2.97–12.77)	ns	4.99 (2.66–8.28)	<0.05∗ ns∗∗
*β*-2 M mg/L	2.53 (1.63–3.71)	2.60 (1.71–3.58)	2.52 (1.51–3.72)	ns	1.75 (1.28–1.96)	<0.001∗ <0.0001∗∗
hsCRP mg/L	4.52 (2.30–7.00)	4.84 (1.50–7.11)	4.54 (2.48–6.94)	ns	—	—
Fibrinogen mg/dL	327.0 (279–392)	328.0 (283–390)	327 (298–372)	ns	—	—

**Table 3:** Serum concentrations of immune activation and inflammatory markers in DCM patients with or without myocarditis according to Dallas criteria and controls, median (25th–75th percentiles).

	Active (A) *n* = 20	Borderline (B) *n* = 70	No MCI (N) *n* = 79	*P* A versus N∗	Controls (C) *n* = 30	*P* A versus C∗ B versus C∗∗ N versus C∗∗∗
NPT nmol/L	5.35 (2.94–12.88)	6.68 (3.96–13.47)	5.06 (2.97–11.38)	ns	4.99 (2.66–8.28)	<0.05∗∗
*β*-2 M mg/L	2.98 (2.56–3.59)	2.63 (1.68–3.71)	2.37 (1.14–3.67)	<0.05∗	1.75 (1.28–1.96)	<0.001∗ <0.001∗∗ <0.05∗∗∗

**Table 4:** Spearman's rank correlation coefficients (*R* and *P*-values) of neopterin, beta-2 microglobulin, and other parameters.

	Neopterin		Beta-2 microglobulin	
	*R* Spearman	*P*	*R* Spearman	*P*
Age	0.11	ns	0.04	ns
Neopterin	X	x	0.30	<0.001
hsCRP	0.23	<0.05	0.15	0.06
Fibrinogen	0.30	<0.01	0.28	<0.01
Macrophages (biopsy)	0.18	<0.05	−0.14	0.09
CD 3 (biopsy)	−0.02	ns	−0.13	ns
EF	−0.17	<0.05	−0.14	0.09
EDV	0.01	ns	0.13	ns
NYHA	0.16	<0.05	0.09	ns
